# Quantification of cytosol and membrane proteins in rumen epithelium of sheep with low or high CH_4_ emission phenotype

**DOI:** 10.1371/journal.pone.0273184

**Published:** 2022-10-18

**Authors:** J. J. Bond, A. J. Donaldson, S. Woodgate, K. S. Kamath, M. J. Mckay, D. Wheeler, D. Tucker, V. H. Oddy

**Affiliations:** 1 NSW Department of Primary Industries, Extensive Livestock Industry Centre, Armidale, NSW, Australia; 2 Department of Molecular Science, Australian Proteome Analysis Facility (APAF), Macquarie University, Sydney, NSW, Australia; 3 NSW Department of Primary Industries, Orange Agricultural Institute, Orange, NSW, Australia; University of Guelph Ontario Agricultural College, CANADA

## Abstract

**Background:**

Ruminant livestock are a major contributor to Australian agricultural sector carbon emissions. Variation in methane (CH_4_) produced from enteric microbial fermentation of feed in the reticulo-rumen of sheep differs with different digestive functions.

**Method:**

We isolated rumen epithelium enzymatically to extract membrane and cytosol proteins from sheep with high (H) and low (L) CH_4_ emission. Protein abundance was quantified using SWATH-mass spectrometry.

**Results:**

The research found differences related to the metabolism of glucose, lactate and processes of cell defence against microbes in sheep from each phenotype. Enzymes in the methylglyoxal pathway, a side path of glycolysis, resulting in D-lactate production, differed in abundance. In the H CH_4_ rumen epithelium the enzyme hydroxyacylglutathione hydrolase (HAGH) was 2.56 fold higher in abundance, whereas in the L CH_4_ epithelium lactate dehydrogenase D (LDHD) was 1.93 fold higher. Malic enzyme 1 which converts D-lactate to pyruvate via the tricarboxylic cycle was 1.57 fold higher in the L CH_4_ phenotype. Other proteins that are known to regulate cell defence against microbes had differential abundance in the epithelium of each phenotype.

**Conclusion:**

Differences in the abundance of enzymes involved in the metabolism of glucose were associated with H and L CH_4_ phenotype sheep. Potentially this represents an opportunity to use protein markers in the rumen epithelium to select low CH_4_ emitting sheep.

## Background

The sheep has a distinct fore-stomach called the reticulo-rumen. It is the site of digestion of a predominantly cellulose and hemi-cellulose rich diet by a diverse variety of Archaea, bacteria, protozoa and fungi. Rumen microbial fermentation supplies the animal with short-chain fatty acids (SCFAs) and microbial proteins as fermentation products. Methane (CH_4_) is formed by methanogens (Archaea) in synergy with microbial fermentation products (such as hydrogen and carbon dioxide) of feed in the rumen. Enteric CH_4_ contributes to atmospheric greenhouse gases (GHG) emitted from livestock. In Australia, enteric CH_4_ contributes to 8–10% of all emissions. The loss of CH_4_ during the fermentation of feed also represents a loss of ingested energy to the animal. There is phenotypic and genetic variation in CH_4_ emission from sheep [1–3]. The basis of some of this variation is difference in the flow of particulate and liquid phase digesta through the reticulo-rumen (mean retention time (MRT) of digesta) [1]. CH_4_ emission from sheep is also positively correlated to dry matter intake (DMI) [4]. A higher proportion of propionate to acetate ratio rumen fluid has been reported [5] in L CH_4_ sheep. Hence it is expected the proportion of metabolites in the rumen of sheep with varying CH_4_ phenotype will differ.

The rumen wall contains three main layers, muscle, connective tissue and a stratified epithelium which has similarities to the skin epithelium [6]. The muscle and connective layer are abundant in collagen, blood proteins, myosin or actin masking the discovery of proteins specific to the epithelium and comprehensive identification of proteins. An in-depth overview of the proteins of isolated rumen epithelium has not been carried out using current proteomic technologies such as high pH HPLC (HpH) separation and SWATH MS quantification.

Experimental procedures to extract rumen epithelium proteins has the potential to greatly improve the annotation of proteins in ruminant genomes. Although the genome of the sheep has been sequenced [7] the annotation of proteins for sheep is poorly described (28,150 UniprotKB proteins of which 473 have been reviewed annotated in Swissprot). Therefore, annotation of sheep proteins and genes relies predominantly on sequence annotations with homology inferred from other species to describe regions or sites of interest in the protein sequence. The current research sought to provide specific detail about the proteins that control the passage of nutrients from the metabolites in the rumen fluid to efflux in the blood. Many of these processes are controlled by integral membrane proteins which are difficult to solubilise and quantify using tandem MS. These include proteins that 1) transport nutrients, 2) control metabolic pathways regulating the fate of nutrients in the rumen epithelium and 3) their subsequent absorption or appearance in the blood.

In the research presented here we extracted cytosol and membrane proteins using an enzymatic procedure [8] from the rumen epithelium of sheep identified as L or H CH_4_ emitting [1] relative to the amount of feed ingested. The isolation procedure was combined with high pH HPLC (HpH) separation and independent data analysis (IDA) tandem mass spectrometry (LC-MS/MS) to form an ion spectral library and then protein abundance was quantified using SWATH MS. Since metabolites in the rumen fluid and gases are in direct contact with the membrane or cytosol proteins of the rumen epithelium we hypothesised there would be quantitative differences in protein abundance related to the transport and metabolism of SCFA’s, amino acids and oligosaccharides between L or H CH_4_ emitting sheep. The research will provide biomarkers to select animals with better nutrient use efficiency and lower CH_4_ emission.

## Methods

### Experimental animals and design

The work was conducted under University of New England Animal Care and Ethics approval (AEC # 14–041). Animals, measurement of phenotypes and methods for sample collection, storage and preparation prior to analysis are described in [1]. Briefly 20 three-year-old ewes (predominantly Merino) were selected for divergence in CH_4_ emission from a group of 64 ewes (63.4 ± 8.52 kg). All sheep with H CH_4_ (28.8 ± 3.26 g/d;n = 10) or L CH_4_ emission (23.5 ± 2.60 g/d;n = 10) were fed the same diet of 50% lucerne chaff and 50% cereal chaff at 1.5 x maintenance requirement for energy. The experimental regime prior to the selection of H or L CH_4_ sheep from the 64 ewes was an incomplete block design experiment with 4 experimental blocks (test periods). Each experimental test period lasted 4 weeks. During test period dry matter intake (DMI) was recorded on the day before and the day of respiration chamber (RC) measurement and liveweight (LWT) was recorded each week. Activities in each test period included 7 days acclimatization of animals to conditions in the animal house, measurement for CH_4_ and carbon dioxide (CO_2_) emissions and methane yield (daily gas emission divided by DMI) in respiration chambers. Then 7 days acclimatization to metabolism cage, after which measurements of feed digestibility (dry matter digestibility; DMD), nitrogen (N) and carbon (C) balance and passage rate of digesta (mean retention time; MRT) were conducted for 7 days. For MRT of solids rumen feed particles were labelled with chromium and rumen liquids measured by a cobalt marker administered as a single dose at the commencement of the measurement period. After metabolism crates the sheep were then returned to their pens. The rumen volume was measured using computer tomography X–ray–CT scan and image analysis as described [1]. Finally, sheep were humanely sacrificed by a qualified operator and a sample (5 cm^2^) of full thickness rumen wall from the ventral sac was removed and frozen at -80°C, as well as a sample of blood collected for serum metabolites frozen and stored at -80°C. A sample of rumen fluid was collected from the rumen contents for metabolite analysis and frozen at -20°C until analysed.

### Isolation of epithelium and extraction of cytosol and membrane proteins

Tissue pieces of ventral rumen wall collected post-mortem from 10 high and 10 low CH_4_ emitting sheep were defrosted. The epithelium proteins were isolated enzymatically from the underlying lamina propria and fractionated according to the method described by [8]. The two fractions extracted were composed of cytosol and membrane proteins. The cytosolic proteins were dialysed against 3 changes of 1% sodium dodecyl cholate (SDC) in 100 mM triethylammonium bicarbonate (TEAB; pH 8.5) over 18 h. The membrane fraction supernatant was dialysed with 3 changes of 1% SDS in 100 mM Tris (pH 8.5), over an 18 h period. The concentration of cytosol and membrane proteins were quantified using the Pierce™ BCA protein assay (Thermo scientific, Rockford, IL, USA) and 2D Quant Kit (GE Healthcare, NJ, USA) respectively.

### Sample preparation for LC-MS/MS analysis

An equal amount of protein (approximately 1 μg) from each sample was used for further testing. SDS in the membrane samples was removed using a detergent removal column (S-Trap™; Profiti, Farmingdale, NY, USA) as per the manufacturer’s instructions and finally eluted using 100 mM Tris-HCl (pH 8.8). Post SDS removal, samples were reduced with 10 mM DTT followed by alkylation with 20 mM iodoacetamide (IAA) in the dark. The reaction was quenched with excess DTT for 15 min. Samples were digested at 37°C, overnight with trypsin at a 1:50 ratio. The digests were quenched with formic acid, and samples were desalted using self-packed SDB-RP StageTips [9].

### Offline high pH (HpH) fractionation by HPLC

A portion of cytosolic and membrane samples was separately pooled and fractionated using HpH chromatography. Briefly, the peptide mixture from the two fractions was resuspended in loading buffer (5 mM ammonia (NH_3_) solution (pH 10.5), separated into 96 fractions using an Agilent 1260 HPLC system. Peptides were separated on a 55 min linear gradient from 3% to 30% acetonitrile in 5 mM NH_3_ solution (pH 10.5) at a flow rate of 0.3 mL/min on an Agilent 300 Extend C18 column (3.5 μm particles, 2.1 mm ID and 150 mm in length). The 96 peptide fractions from each fraction were consolidated into 17 fractions to adequately sample the peptides separated by HpH in the chromatographic profile. Samples were subsequently dried in a vacuum centrifuge and reconstituted in 2% acetonitrile, 0.1% formic acid for LC-MS/MS processing.

### LC-MS/MS and data acquisition

Individual samples from each sheep and each fraction were analyzed on a TripleTOF 6600 mass spectrometer (SCIEX) in two stages: Information-Dependent Acquisition-MS (IDA-MS) analysis of HpH fractionated peptides for spectral library generation, followed by Data-Independent Acquisition-MS (DIA-MS) analysis of individual samples using SWATH-MS procedure for label-free quantification [9].

Nanoflow LC-MS/MS analysis was carried out in positive ion mode using a Triple TOF 6600 mass spectrometer (SCIEX) equipped with an Eksigent Ultra nanoLC system (Eksigent) and nanoflex cHiPLC module (SCIEX). Peptides (10μl, approx. 2 μg) were desalted with 2% acetonitrile, 0.1% formic acid using a C18 trap (Halo-C18, 160 Å, 2.7 μm, 200 μm x 2 cm) for both IDA and DIA-MS experiments.

For IDA, peptides were eluted from a trap column and separated on a cHiPLC C18 column (15 cm x 200 μm, 3 μm, ChromXP C18-CL, 120 Å, 25°C, SCIEX) using a linear solvent gradient from 2% acetonitrile (0.1% formic acid) to 35% mobile phase B (B: 99.9% acetonitrile, 0.1% formic acid) at 600 nL/min over 120 min. For SWATH-MS, data was acquired using a 60 min LC gradient (5–35% mobile phase B) at 600 nl/min. Liquid chromatography eluent was subjected to positive ion nanoflow electrospray MS analysis (spray voltage 2.5 kV, curtain gas 25) using a nanospray III source (SCIEX) and an uncoated PicoTip Emitter (New Objective, USA). First a TOFMS survey scan (m/z 350–1500, 250 ms) was conducted followed by MS/MS analysis (2+ to 4+; 100 ms each, m/z 100–1800) of the top 20 most intense precursor ions with a dynamic exclusion time of 30 s.

For SWATH experiments, individual samples were analyzed in DIA-MS mode using variable m/z windows (100 in total) determined based on precursor m/z densities from IDA data. First, a TOFMS survey scan was acquired (m/ z 350–1500) followed by 100 SWATH-MS2 scans (m/ z 350–1500). Each SWATH-MS2 scan used rolling collision energy and CE spread of 5. SWATH experiments for individual samples were acquired in a randomized order with one blank injection acquired between each sample.

### Bioinformatic analysis to predict protein subcellular location and biological function

Protein identifications with 2 peptides and a confident protein score (P <0.05) from the HpH fractionation and IDA-MS were used to assign subcellular localization. Using the top score given by WoLF PSORT [10] (www.genscript.com/wolf-psort.html) proteins were categorized into 8 locations. Membrane proteins were predicted using transmembrane helical Markov model (TMHMM) [11] (www.cbs.dtu.dk/services/TMHMM/). Proteins in the solute carrier protein (SLC) and ATP-binding cassette (ABC) transporter families were identified according to gene and protein name. We also used website gene names (www.genenames.org/data) to characterise the subcellular location of the transporter and the type of substrate they transport.

For proteins annotated as ‘uncharacterised’ in figures and tables in the manuscript a BLAST protein homology search was carried out using the Ensembl or uniport accession code in uniprotKB (www.uniprot.org). The accession code page contains the sequence and a link to BLAST. BLASTp results against uniprotkb_Swissprot reference proteomes and an identity sequence match of >95.5% to human, bovine (cattle) or caprine (goat) proteome annotation was accepted as the protein name.

### Rumen fluid and blood metabolite quantitation

Fifty ml of rumen liquid was obtained from rumen contents post-mortem from each sheep matching the H and L CH_4_ sheep numbers used for protein analysis, approximately 3 h after morning fed was offered. Samples were frozen at -20°C until analysis for rumen fluid metabolites by nuclear magnetic resonance spectrometer (NMR). Prior to NMR samples were thawed, then centrifuged (Beckman microfuge 16; 16 000 x *g*) and the supernatant passed through a 0.22 μm filter. For more details of the method please refer to [12].

^1^H NMR spectra were acquired at 298K in 3 mm tubes on a Bruker Avance 900 NMR spectrometer with CryoProbe using a SampleJet (96 tube racks) for sample introduction. Samples were maintained at 4°C in the SampleJet prior to introduction into the probe and an equilibration time of 6 min was allowed before commencement of acquisition. Standard Bruker pulse sequences were used. NMR spectra were processed with Topspin 3.2 software, using multiplication by a sine bell, shifted by 90°, prior to Fourier transformation and manual phase correction. Spectra were referenced to internal 4,4‑dimethyl‑4‑silapentane-1-sulfonic acid (DSS) (δ0.0). Metabolite concentrations were determined by integration relative to the integral of the internal standard, difluorotrimethylsilylmethylphosphonic acid (DFTMP; 500 μM). Metabolites were identified by 2D NMR spectroscopy (total correlation spectroscopy, correlation spectroscopy, heteronuclear single quantum correlation and heteronuclear multi-bond correlation) and their identity was confirmed by comparison with literature values for ^1^H and ^13^C NMR shifts.

Blood samples were collected post-mortem matching the H (n = 8) and L CH_4_ (n = 8) sheep number used for proteomic analysis of tissue. Blood beta hydroxybutyrate (BHB) used as a measure of low energy levels in the form of a ketone body in the blood was assayed as described by [13]. Blood glucose concentration was analyzed by the hexokinase method (Roche Diagnostics Ltd). D -Lactate in serum was measured utilizing enzymes D—Lactate dehydrogenase and glutamate-pyruvate transaminase [14]. Similarly, L-lactate in serum was measured by an enzymatic assay (Regional Laboratory Services, Benalla, Victoria, Australia; https://www.regionallabservices.com.au/).

### Data analysis

For mass spectrometry data a spectra library was generated by performing a combined data search of all the IDA-MS data using the Paragon algorithm (SCIEX) in ProteinPilot (Version 5.0, SCIEX) in thorough ID mode with FDR calculation enabled and allowing biological modification. All the MS/MS spectra from IDA experiments were searched against a reference database for the Ovine proteome (Oar v4. Ensembl, May 2017). Protein identifications were accepted with carbamidomethylation of cysteine residues, only proteins identified with 2 or more peptides and a Protscore *P* <0.05.

**Fig 1:**
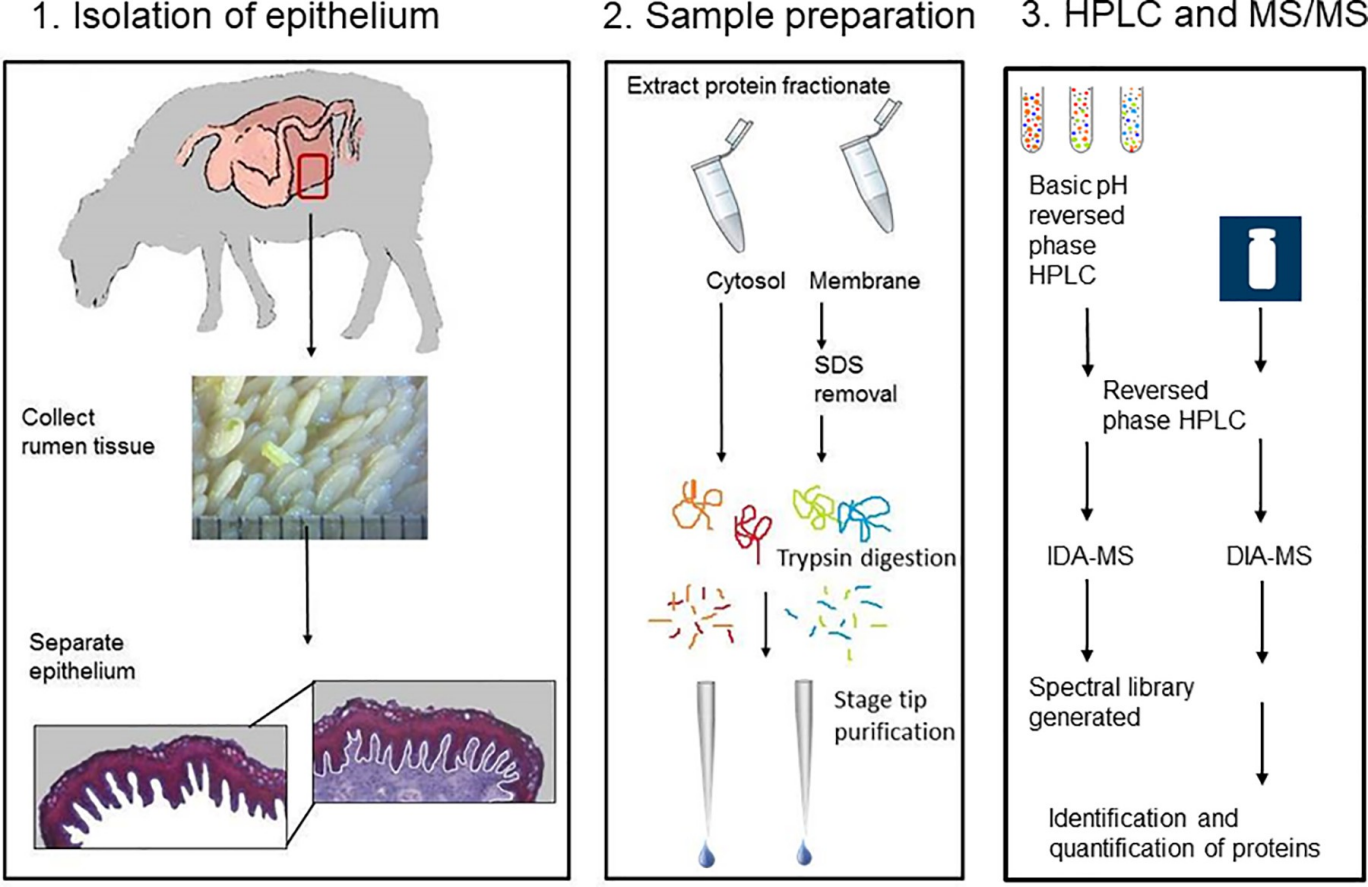
Workflow of the epithelium proteome analysis 1). Whole depth rumen tissue (n = 20) were collected, treated with enzyme and the epithelium isolated by microdissection. 2). Sample preparation; Each epithelial tissue sample was homogenised and fractionated into a cytosol and membrane fraction (n = 20/fraction), the protein extracts dialysed and SDS removed prior to trypsin digestion. 3). High pH (HpH) fractionation by HPLC, followed by LC-MS/MS analysis of the peptides in Independent Data Acquisition (IDA-MS) mode. 4) Data-Independent Acquisition-MS (DIA-MS) analysis of individual samples using SWATH-MS procedure for label-free quantification. Protein identifications were made using Ensembl ovine database.

For SWATH quantitation, consolidated ProteinPilot IDA search results were imported into PeakView 2.1 with SWATH 2.0 MicroApp (SCIEX) and used as a spectral library. Retention times for all SWATH files were aligned using linear regression model using endogenous peptides across the elution profile. The top 6 most intense fragment ions for each peptide were extracted from the SWATH data using a maximum number of peptides of 100, 75 ppm mass tolerance, peptide confidence threshold of ≥ 0.99, and a 5 min retention time extraction window. After data processing, peptides with confidence > 99% and FDR < 1% (based on chromatographic feature after fragment extraction) were used for quantitation. The extracted peak areas were exported into Excel for further statistical analysis. Differentially expressed proteins were determined by pairwise comparisons of pairs of samples using t-tests on log-transformed normalized protein peak areas; peptide level t-tests were also carried out [15]. Proteins were deemed to be differentially expressed if the ANOVA p value was less than 0.05, and the protein fold change exceeded 1.5 [16, 17]. Proteins identified with 1 or more peptides were retained in the proteins accepted with differential abundance capable of discriminating between phenotypic group for CH_4_ emission. The workflow described in the above steps are summarised in Fig 1. The search results are available at proteomics identification database PRIDE (https://www.ebi.ac.uk/pride/archive/, PRIDE ID: PXD026538) and S1 Table.

Phenotypic trait data were analyzed using a general linear model (www.minitab.com; v18) [18]. Variable effects tested were CH_4_, CO_2_, MY, rumen volume, N and C balance, DMD, MRT with fixed effects of experimental test period (n = 4 data taken from incomplete block design experiment) and phenotype (L or H CH_4_) adjusted for covariable effects of DMI and liveweight. Rumen fluid metabolite and blood metabolite data were analysed using a general linear model with metabolite as variable effect and fixed effect of phenotype adjusted for a covariable effect of liveweight.

## Results

### Methane emission and digestive parameters in sheep selected for L or H CH_4_ group

Since digestive function varies between H or L CH_4_ phenotype a summary describing the relevant digestive parameters are shown in Table 1. Daily CH_4_ (g/d; *P*<0.001), CO_2_ (g/d; *P* = 0.01) emission and methane yield (MY g CH_4_/kg DMI; *P* = 0.003) were significantly lower in the L CH_4_ sheep than H CH_4_ sheep when adjusted for liveweight and DMI. Mean retention time (MRT) of rumen feed particles (*P* = 0.03) and of rumen liquids (*P* = 0.004) was also significantly less in the L CH_4_ sheep than H CH_4_ sheep. That is the passage rate of solid and liquid rumen contents was faster in the L CH_4_ sheep. There was no statistical difference in rumen volume, N and C balance or DMD between sheep in the two CH_4_ phenotypes.

**Table 1:** Descriptive statistics (mean ± standard deviation of the mean) for phenotypic traits related to CH_4_ emission in high (H) or low (L) emitting sheep; liveweight (kg), dry matter intake (DMI), daily CH_4_ (g/d), CO_2_ (g/d), methane yield (MY), rumen volume (cm^3^), nitrogen (N) balance (g/d), carbon (C) balance (g/d), dry matter digestibility (DMD; g/kg), mean retention time (MRT) rumen particles and MRT liquids (/d). P values represent statistical differences in the variables related to the high (H; n = 10) and low (L; n = 10) CH_4_ groups.

Variable	H CH_4_	L CH_4_	P value CH_4_ phenotype
**Liveweight (kg)**	66.3 ± 9.25	63.0 ± 5.24	ns
**DMI (kg)**	1.46 ± 0.185	1.34 ± 0.150	ns
**CH**_**4**_ **(g/d)**	28.8 ± 3.26	23.5 ± 2.60	0.001
**CO**_**2**_ **(g/d)**	1024 ± 106.7	904.7 ± 77.3	0.01
**MY (g CH**_**4**_ **/ kg DMI)**	19.9 ± 1.12	17.8 ± 1.65	0.003
**Rumen Volume (cm** ^**3**^ **)**	9813 ± 1515	8581 ± 1877	ns
**N balance (g/d)**	14.5 ± 3.26	13.7 ± 3.57	ns
**C balance (g/d)**	367.6 ± 42.90	358.0 ± 48.20	ns
**DMD (g/kg)**	642.4 ± 21.44	661.1 ± 26.08	ns
**MRT rumen particles (d** ^**-1**^ **)**	1.04 ± 0.091	0.90 ± 0.160	0.029
**MRT rumen liquids (d** ^**-1**^ **)**	0.70 ± 0.057	0.59 ± 0.081	0.004

### Protein identification and quantitation of rumen epithelium proteins

Following the identification of the proteins in the cytosol and membrane fraction by HpH fractionation and IDA-MS the lists were combined to obtain the total number of proteins identified (n = 2767; S1 Table). Notably, 220 proteins were unique to the cytosol fraction, 1963 unique to the membrane fraction and 583 found in both fractions (peptides matched 2, protscore *P*<0.05, peptide FDR<1%). Selective enrichment of rumen tissues [8], initial solubilisation using detergent (SDS) and HpH fractionation of proteins enabled the in-depth characterisation of proteins.

### Assignment of proteins to subcellular compartment

**Fig 2:**
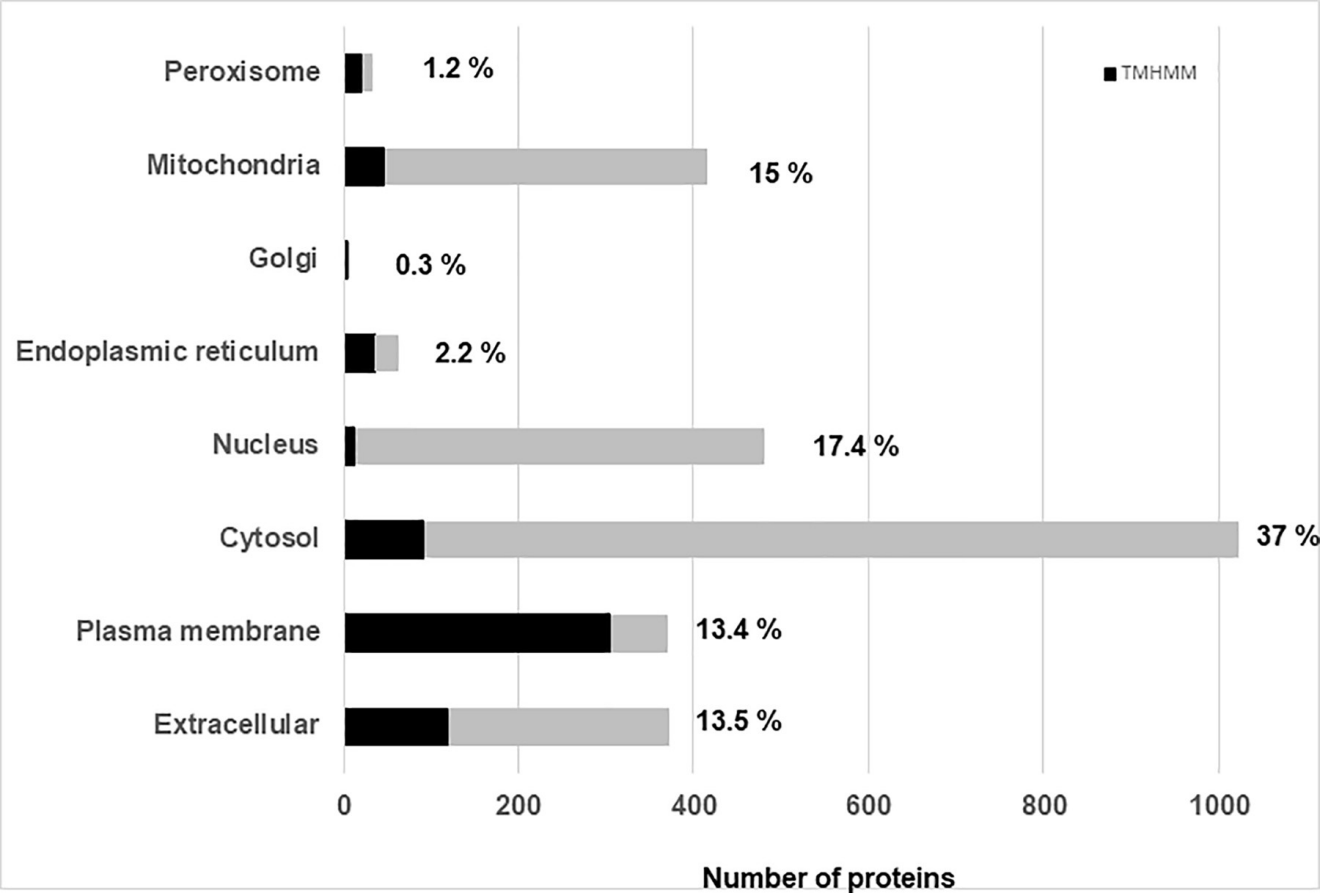
Number of proteins identified using HpH fractionantion and IDA-MS in subcellular locations predicted by WolF PSORT. The proportion of proteins predicted to have more than 1 membrane domain (TMHMM; black), is incorporated into the count for each subcellular category. The % of the total number of proteins in each category is shown beside the respective bar.

To expand the knowledge of the location and function of the proteins identified subcellular location predictions by WoLF PSORT [10] revealed a large proportion (% of total) of proteins were localised in the cytosol (*n* = 1023; 37%), nucleus (*n* = 481; 17.4%), mitochondria (*n* = 415; 15%), plasma membrane (*n* = 371; 13.4%) or extracellular (*n* = 373; 13.5% Fig 2) compartment of the cell. Smaller proportions were assigned to the endoplasmic reticulum (*n* = 23; 2.2%), peroxisome (*n* = 34; 1.2%) or golgi (*n* = 7; 0.3%).

Of all the proteins identified 23% were predicted membrane proteins with at least one transmembrane domain (TMHMM; S1 Table). The plasma membrane category had a majority of proteins with multiple transmembrane domains (n = 219 >1 TMHMM). Those assigned to the cytoplasm or extracellular compartment were largely single transmembrane domain proteins (1 TMHMM).

### Identification of proteins that transport nutrients and ions in the rumen epithelium

Two main families of transporters were identified, the solute carrier (SLC) family and ATP-binding cassette (ABC) transporters (S1 Table; transporters). Most members of the SLC group had >3 TMHMM indicating they are integral plasma membrane proteins.

Those that transport carbohydrates were 2 different glucose transporters SLC2A1 (GLUT1) and SLC5A1 as well as several SLC35 family transporters which exchange UDP-sugars. We also detected amino acid transporters, including, SLC1A4, SLC1A5, SLC3A2 (neutral amino acids) and SLC18A1 (amine). Lipid transporters found were SLC16A1 (SCFA/lactate), SLC26A3 (SCFA/HCO_3_^-^), SLC27A1 (LCFA), SLC27A2 and SLCA4 (fatty acid transport). Those that transport metabolic intermediates were SLC14A1 (urea), SLC44A1, SLC44A2 and SLC44A3 (choline). Eight ABC transporters identified all had multiple transmembrane domains indicating they were core components of multiprotein complexes on the cell surface. ABCA12 and ABCA13 transport fatty acids involved in the formation of the lipid barrier.

Many of the transporters identified regulate cell ion homeostasis through transport of inorganic ions across the plasma membrane. They include (n = 43 SLC) SLC9A3 (Na^+^/H^+^), SLC12 A4 and SLC12A6 (K^+^/Cl^-^), SLC22A18 (cation), SLC26A2 (anion/sulfate), SLC30A1 and SLC30A7 (Zn^2+^), SLC40A1 (H^+^/metal). Anions transporters identified were the core maxi-Cl channel transporter SLCO2A1 which is permeable to Cl^-^ and SCFA anions [19]. A volume-sensitive outwardly rectifying anion channel (VSOR; LRRC8E) and chloride intracellular channel proteins (CLIC1 and CLIC4), members of the CLC family of chloride gated channels (CLCA2, CLCN1, 2, 3) and the anoctamin family (ANO 6, 9,10) were identified. Three subunits of ATP1 (Na^+^/K^+^), three subunits of ATP2 (Ca^2+^), and ATP8 or ATP9 that transport phospholipids were also found. The ATP1 protein is a major driver of the concentration gradient of solutes in the epithelium at the basal level.

Despite the comprehensive identification of these transporter proteins only SLC40A1 had higher abundance in the in the H CH_4_ (FC 3.72) phenotype compared to the L CH_4_ emitting phenotype.

### Quantitative differences in epithelium protein abundance in H or L CH_4_ rumen epithelium

In the cytosol fraction we found 12 proteins which had a fold change >1.5 in the H and L CH_4_ emitting ewes and were significantly different using ANOVA (*P*<0.05; S1 Table; cytosol SWATH). Eight proteins (Cysteinyl-tRNA synthetase; CARS, Lactate dehydrogenase D;LDHD, Terpene cyclase/mutase family member; LSS, Malic enzyme; ME1, Nuclear receptor coactivator; NCOA2, Proteasome subunit beta type;PSMB5, Myoferlin; MYOF; Moesin; MSN) had significantly higher fold change in the L CH_4_ group compared to the H CH_4_ group.

4 proteins (Hydroxyacylglutathione hydrolase; HAGH, Galectin 3 binding protein; LGALS3BP, Actin-related protein 2/3 complex subunit, Hydroxypyruvate isomerase; HYI) had higher abundance in the H CH_4_ group compared to the L CH_4_ group.

In the membrane fraction we found 44 proteins which had a fold change >1.5 and were significantly different using ANOVA (*P<*0.05; S1 Table; membrane SWATH) in the H and L CH_4_ emitting ewes. 21 proteins had significant increase in abundance in the L CH_4_ group (RETN, Azurocidin 1; AZU1, Polyribonucleotide nucleotidyltransferase 1; PNPT1, ATP-dependent (S)-NAD(P)H-hydrate dehydratase; CARKD, Cytochrome c; CYCS, 5 uncharacterised proteins, Protein disulfide-isomerase; PDIA4, Immunoglobulin heavy constant mu; IGHM, ADP dependent glucokinase; ADPGK, Fibrinogen gamma chain; FGG, Pterin-4 alpha-carbinolamine dehydratase 2; PCBD2, Pitrilysin metallopeptidase 1; PITRM1, AP complex subunit beta; AP1B1, Pre-mRNA processing factor 40 homolog A; PRPF40A, FAST kinase domains 2; FASTKD2, DCTN1, Myeloperoxidase; MPO) compared to the H CH_4_ phenotype. 23 proteins had significant increase in abundance in the H CH_4_ group (Solute carrier family 40 protein; SLC40A1, Hydroxysteroid 17-beta dehydrogenase 11; HSD17B11, Golgi SNAP receptor complex member 1; GOSR1, Ribosomal oxygenase 1; RIOX1, Nephroblastoma overexpressed; NOV, Elongation factor 1-alpha; EEF1A1, Mitochondrial ribosomal protein L48; MRPL48, Serpin family B member 12; SerpinB12, Glutathione S-transferase; GSTM3, RHOA, Peroxisomal biogenesis factor 14; PEX14, Beta-1,3-galactosyl-O-glycosyl-glycoprotein beta-1,6-N-acetylglucosaminyltransferase 3; GCNT3, SEC23 interacting protein; SEC23IP, Nitric oxide synthase; NOS2, DEK proto-oncogene; DEK, Spectrin repeat containing nuclear envelope protein 2; SYNE2, TATA-box binding protein associated factor 4b; TAF4B, Signal transducer and activator of transcription; STAT1, Deoxyribonuclease 1 like 1; DNASE1L1, Interleukin 1 receptor type 2; IL1R2, RPL34, LEM domain containing 3; LEMD3 and 1 uncharacterised protein) compared to the L CH_4_ phenotype. Table 2 shows a subset of these proteins which have significant FC in protein abundance, 3 of which can be related to metabolic pathways and 3 proteins to cell defense to microbes explained in the following sections.

**Table 2:** Rumen epithelium proteins quantified with significant differences (*P*<0.05; fold change (FC) >1.5). Fold change in protein is greater in the L compared to the H CH_4_ emission phenotype A) L CH_4_ > H CH_4_ protein FC. Where the protein abundance is higher in the H compared to the L CH_4_ emitting phenotype B) H CH_4_ > L CH_4_ protein FC. Accession Ensembl (*Ovis aries* database), Uniprot accession code, protein and gene name from gene ontology data, number of peptides matched to the sequence and fold change are shown. Subcellular location was assigned using WoLF PSORT. For a complete list of proteins with significant differences see S1 Table.

Protein	Entry	Protein names	Gene names	location	No. Peptides matched	FC
**A)** L CH_4_ > H CH_4_ protein FC						
ENSOARP00000006849.1	W5P8U2	Lactate dehydrogenase D	LDHD	mitochondria	1	2.20
ENSOARP00000008043.1	W5PC82	Malic enzyme	ME1	cytosol	5	1.57
ENSOARP00000002425.1	W5NW78	Resistin	RETN	extracellular	2	2.37
ENSOARP00000004349.1	W5P1Q0	AP complex subunit beta	AP1B1	cytosol	2	1.92
ENSOARP00000010314.1	W5PIP4	Azurocidin 1	AZU1	cytosol	1	2.48
ENSOARP00000009967.1	W5PHQ0	Myeloperoxidase	MPO	cytosol	1	3.50
ENSOARP00000020820.1	W5QDM2	Proteasome subunit beta	PSMB5	cytosol	2	1.60
ENSOARP00000022795.1	P62896	Cytochrome c	CYC	cytosol	4	1.57
ENSOARP00000003016.1	W5NXW9	Immunoglobulin heavy constant mu	IGHM	cytosol	4	1.63
**B) H CH**_**4**_ **> L CH**_**4**_ **FC**						
ENSOARP00000017830.1	W5Q540	Hydroxyacylglutathione hydrolase	HAGH	cytosol	1	2.56
ENSOARP00000017599.1	W5Q4F9	Solute carrier family 40 protein	SLC40A1	Plasma membrane	1	3.72
ENSOARP00000020752.1	W5QDF4	Glutathione S-transferase (EC 2.5.1.18)	GSTM3	cytosol	2	1.77
ENSOARP00000017986.1	W5Q5J6	Nitric oxide synthase (EC 1.14.13.39)	NOS2	cytosol	3	1.67
ENSOARP00000014105.1	W5PUH3	Interleukin 1 receptor type 2	IL1R2	Plasma membrane	2	1.52

### Metabolic pathways associated with significant fold changes in epithelium proteins in H or L CH_4_ emitting sheep

**Fig 3:**
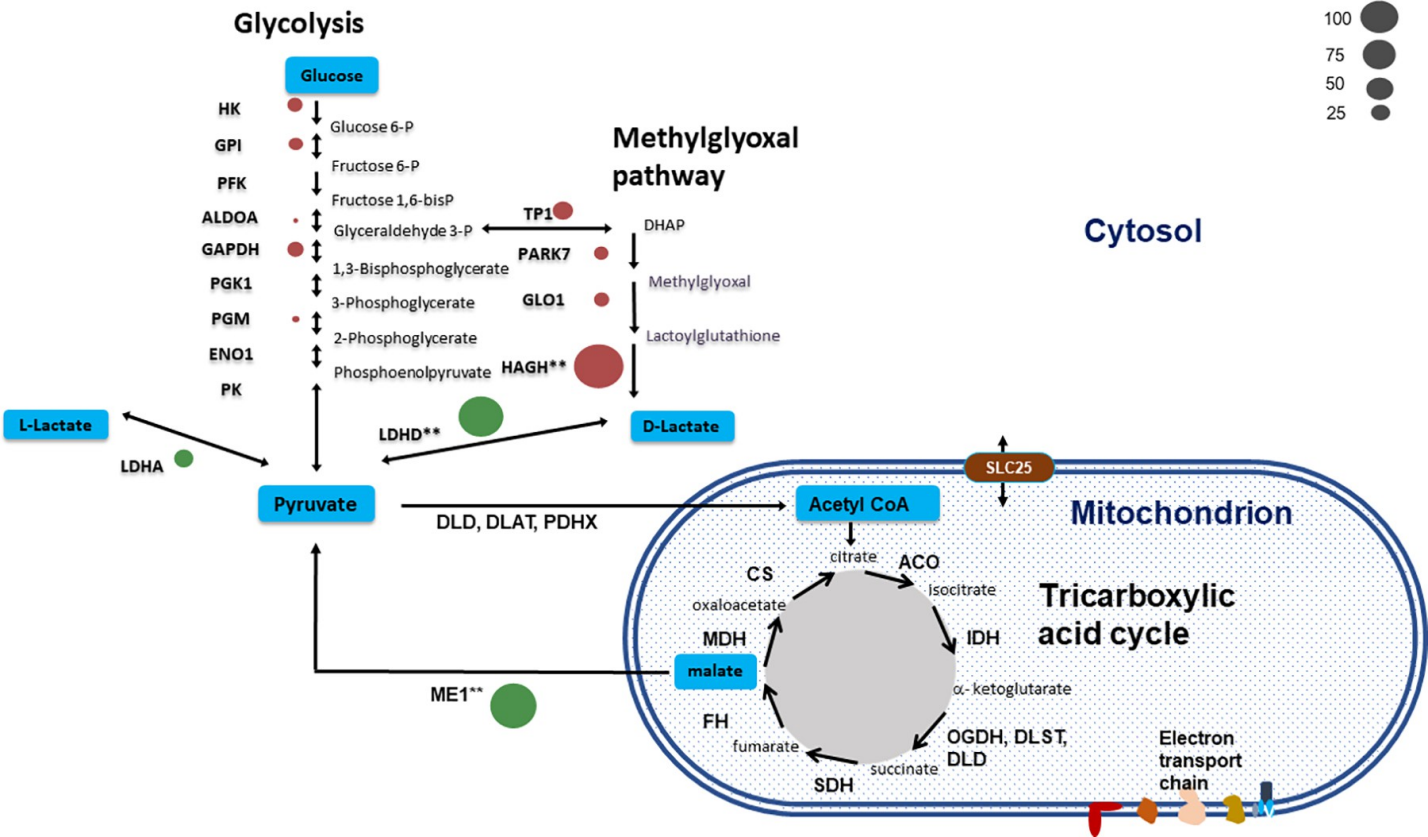
A diagram representing the enzymes involved in the 11 steps of the glycolytic and methylglyoxal pathway and tricarboxylic cycle found in the rumen epithelium of sheep. Significant fold change in proteins (*P*<0.05) is marked with a double asterisk (**). Fold change is represented by bubble size and an increase in the abundance of an enzyme in the low CH_4_ group shown by a green bubble, whereas increase in the abundance of an enzyme in the high CH_4_ group shown by red bubble. Abbreviations of protein names: HK; hexokinase, GPI; glucose-6-phosphate isomerase, PFK; phosphofructokinase-1, ALDOA; fructose-bisphosphate aldolase, GAPDH; glyceraldehyde-3-phosphate dehydrogenase, PGK1; phosphoglycerate kinase, PGM; phosphoglycerate mutase, ENO1; enolase 1, PK; pyruvate kinase, LDHA; L-lactate dehydrogenase LDHD; D-lactate dehydrogenase, TPI1; triosephosphate isomerase, PCK2; pyruvate carboxykinase -2, P; phosphate. For enzymes involved in the TCA pathway and associated pyruvate or malate cycling. Abbreviations are DLAT; acetyltransferase, DLD; dihydrolipoyl dehydrogenase, PDHX; dihydrolipoamide acetyltransferase, CS; citrate synthase, ACO; aconitase, IDH; isocitrate dehydrogenase, OGDH; oxoglutarate dehydrogenase, DLST; Dihydrolipoamide S-succinyltransferase, FH; fumarate hydratase, MDH; malate dehydrogenase, ME1; malic acid enzyme, PCK2; pyruvate carboxykinase -2. ALAT; alanine aminotransferase; PAG; phosphate-activated glutaminase.

Enzymes that had significant fold change were associated with two major energy utilising pathways, glycolysis and the tricarboxylic acid cycle. The 11 enzymes involved in the glycolysis pathway versus the reverse process storing glucose as glycogen were identified in tissue from each CH_4_ phenotype (Fig 3). A side pathway of glycolysis, the methylglyoxal (MGO) pathway was found in which 2 proteins had higher fold change between CH_4_ group. There are several steps in the MGO pathway the first of which methylglyoxal synthase converts dihydroxyacetone phosphate (DHAP) via triosephosphate isomerase (TPI) to methylglyoxal by-passing glycolysis. The first two enzymes in the pathway methylglyoxal synthase (PARK7) and glyoxylase I (GLO1) or lactoylglutathione lyase were identified. In the next step lactoylglutathione is converted to D-lactate and glutathione by glyoxylase II also called hydroxyacylglutathione hydrolase (HAGH). In the H CH_4_ epithelium HAGH was 2.56 fold higher than in the L CH_4_ epithelium. MGO is a toxic compound which requires rapid detoxification by the MGO pathway. Associated with the higher abundance of HAGH was a higher FC (1.77 FC) in the H compared to the L CH_4_ of enzyme S-transferase (GSTM3). The enzyme produces GSH and functions to detoxify compounds, such as products of oxidative stress, by conjugation with glutathione.

In the last step of the MGO pathway methylglyoxal is converted to D-lactate, which may be converted to pyruvate by D-lactate dehydrogenase. Lactate dehydrogenase D (LDHD) was 1.9 fold higher in the L CH_4_ epithelium than the H CH_4_ sheep. Malic enzyme (ME1) was 1.57 fold higher in the L CH_4_ group and is known to play an important role in the conversion of malate then to pyruvate through the TCA cycle (Fig 3).

Also related to regulation of insulin sensitivity and glucose uptake by cells we found resistin (RETN) was 2.37 fold higher in the L CH_4_ phenotype.

### Differential abundance of proteins involved in cell defense against microbes in the epithelium of L or H CH_4_ sheep

In the H CH_4_ sheep (Table 2), we found increased abundance of nitric oxide synthase (NOS2) of the peroxisome [20]. Galectin 3 binding protein (LGALS3BP; FC 1.7) was significantly higher in the H than L CH_4_ sheep. Also interleukin 1 receptor (IL1 R2; 1.52 FC) was 1.52 fold higher in the H than L CH_4_ sheep.

In the L CH_4_ sheep myleoperoxidase (MPO) was 3.5 fold higher than in H CH_4_ sheep epithelium. AP complex subunit beta (AP1B1) had a greater fold change (1.9 FC) in the L than the H CH_4_ sheep. Azurocidin 1 (AZU1) a known anti-microbial, had a 2.48 higher abundance in the L than the H CH_4_ sheep.

### Rumen fluid metabolites H or L CH_4_ phenotype

Since we expected different metabolic conditions in the rumen of each CH_4_ phenotype to affect protein expression in the cytosol or membrane proteins of the rumen epithelium in contact with these conditions, a subset of the metabolites identified and quantified in the rumen fluid (3h after feeding) collected post-mortem are shown in Table 3 and S2 Table. These include 13 amino acids (AA), 3 SCFA’s and 6 carbohydrates. The main metabolites of microbial fermentation of complex carbohydrates in the rumen are acetate, butyrate and propionate. Including glucose all had a concentration above 3.5 μM in the rumen fluid of sheep feed the same fibrous 1.5 x maintenance energy level diet. There were no significant differences in metabolite concentration adjusted for liveweight found between L or H CH_4_ phenotype at this sampling time point.

**Table 3:** Metabolites concentration (μM) quantified using NMR in rumen fluid collected post-mortem approximately 3 h after feeding in L or H CH_4_ emitting sheep (n = 10/ CH_4_ group, mean ± standard deviation). *P*>0.05 (ns) for all measures in the table.

Metabolite	L CH_4_	H CH_4_
**Carbohydrates**		
**maltose**	402 ± 174.3	367 ± 182.6
**b-ribose**	184 ± 71.3	171 ± 76.5
**xylose**	19 ± 10.3	17 ± 10.7
**lactate**	292 ± 53.5	279 ± 59.3
**Dihydroxy acetone (DHA)**	40 ± 10.2	45 ± 12.9
**glucose**	4015 ± 1683	3697 ± 1871
**Short chain fatty acids**		
**acetate**	51528 ± 5978	53596 ± 7589
**propionate**	14562 ± 1894	15464 ± 3034
**butyrate**	9402 ± 1109	9685 ± 1511
**Amino acids**		
**tryptophan**	54 ± 14.3	49 ± 13.2
**phenyl alanine**	130 ± 37.2	119 ± 39.5
**tyrosine**	100 ± 35.1	91 ± 32.4
**proline**	642 ± 236.0	626 ± 276.8
**glycine**	545 ± 210.9	507 ± 175.9
**lysine**	1050 + 234.2	850 + 195.6
**aspartate**	825 ± 175.3	843 ± 202.4
**glutamate**	825 ± 230.6	783 ± 226.2
**alanine**	704 ± 221.7	670 ± 242.5
**isoleucine**	252 ± 78.2	226 ± 77.4
**valine**	379 ± 98.0	357 ± 105.6
**leucine**	346 ± 85.6	319 ± 92.8
**threonine**	169 +108.8	189 + 126.1

### Metabolites in the blood

Serum samples were analysed for beta hydroxy butyrate (BHB), glucose and D-lactate and L-lactate. (Table 4; S2 Table). There were no significant effects liveweight. Glucose (*P* = 0.02) and L-lactate was significantly higher (*P* = 0.006) in the L CH_4_ phenotype.

**Table 4:** Blood metabolites (mM) of sheep related to quantitative differences in proteins of rumen epithelium in L (n = 8) or H (n = 8) CH_4_ emitting sheep. (BHB; Beta-hydroxybutyrate).

	L CH_4_	H CH_4_	*P* value
**Glucose**	5.89	3.96	0.02
**L-lactate**	13.4 ± 1.65	6.4 ± 0.74	0.006
**D-lactate**	0.06 ± 0.052	0.03 ± 0.016	ns
**BHB**	0.28 ± 0.029	0.30 ± 0.053	ns

## Discussion

We have presented a comprehensive proteomic landscape of the ovine rumen epithelium. Since our first attempts to profile proteins of enzymatically isolated rumen epithelium (*n* = 570 proteins) [8], we have been able to substantially increase the depth of proteome coverage by 5-fold (*n* = 2767 proteins).

Enrichment of membrane proteins and the high coverage of proteins identified in the membrane fraction (23% with TMHMM) allowed us to identify many nutrient transporters previously only reported at the transcript level. Compared to our previous attempt to identify these protein transporters [8] using gel-based separation we increased the number found 3.5 fold from 25 to 92 using the current proteomic approach. Two families of transporter proteins were well represented including the solute carrier family (SLC) and ATP binding cassette family (ATP). A transporter that has not been detected in rumen epithelium, SLCO2A1 protein, was found in the present study. Electrophysiology studies [21, 22] point to the existence of a maxi-Cl channel permeable to Cl^-^ and SCFAs. The identity of molecular candidates for the channel has been debated, but recent evidence clearly defines the core of the maxi-Cl channel as organic anion transporter SLCO2A1 [19]. We confirm the presence of SLCO2A1 protein containing 11 transmembrane domains in our rumen epithelium tissue representing an alternative route to transport SCFAs than the well described SLC16A1 (MCT-1) [23, 24]. Other transporters of interest identified were, subunit LRRC8E of the volume-sensitive outwardly rectifying anion channel family (VSOR) [25, 26] which plays a role in regulating cell volume. Recently the transient receptor potential (TRP) channel TRPV3 has been shown to be permeable to Na^+^, Ca^2+^, NH^4+^ [27]. We identified a related protein TRPM4 which is a Ca^2+^-activated nonselective cation channel mediating cell membrane depolarization [28].

Supporting information to outline the metabolites identified in the rumen fluid or blood and changes in the rumen epithelium proteins involved in key metabolic pathways has helped us to track the metabolic fate of key nutrients during their passage through the epithelium. In ruminants, the main products of fermentation of dietary fibre are SCFAs, (acetate, propionate and butyrate) which account for more than 70% of the animal’s caloric intake [29]. In this review it states glucose was rarely detected in rumen fluid or intestinal fluid. However, using modern techniques like NMR detected concentrations of 4.0 mM in L CH_4_ rumen fluid around 3 h after being fed a fibrous diet. In a similar study in dairy cows by Saleem et al. [30] using NMR found the concentration of glucose was 0.5 mM (prior to feeding a diet with increasing amounts of barley grain). Glucose, other mono- or oligosaccharides and SCFA’s may be absorbed from the rumen fluid by plasma membrane transporters in the epithelium. Two main glucose transporter families, SLC2A (GLUT) and SLC5A (Na^+^/glucose symporter), have been previously reported in the bovine rumen [31]. Similarly, Aschenbach *et al*. [32] demonstrated in sheep rumen epithelium gene expression of SLC5A1 (SGLT1) and its transport of D-glucose from the lumen to the blood *in vitro*. We identified SLC2A1 (GLUT1) and the SLC5A1 protein in the rumen epithelium proteins and associated changes in epithelium enzymes related to glucose metabolism. Coincidently, we detected higher blood glucose in the L CH_4_ serum. Collectively these findings indicate glucose is available in the rumen fluid and may be transported into the rumen epithelium but these concentrations may fluctuate with feeding and pattern of fermentation.

The time of sampling the rumen fluid coincides with peak methane emission [1] in the rumen. It is surprising we did not detect a significant difference between any of the metabolites measured in the rumen fluid between L and H CH_4_ phenotype. Although this might have been related to fermentation pattern or other related factors. These include the L CH_4_ sheep phenotype being associated with shorter MRT of digesta [1, 33, 34], higher proportion of propionate to acetate ratio rumen fluid [5] and the rumen microbiome being enriched by lactic acid forming bacteria such as *Sharpea* [35] of L CH_4_ yield sheep.

Together these results show glucose, simple saccharides and SCFA’s may be utilised to maintain rumen epithelium cell energy homeostasis and to support their high rate of cell division and protein synthesis. The differences in the abundance of enzymes at the end of the glycolytic pathway or methylglyoxal pathway (MGO) in the H or L CH_4_ sheep epithelium found reflects different mechanisms of energy use or detoxification occurred between the CH_4_ phenotypes.

To our knowledge the MGO pathway has not been reported in sheep rumen epithelium in previous research, although the genes for the enzymes involved exist in the ovine, caprine and bovine genome. It was once thought D-lactate only occurred from exogenous sources in ruminant epithelium from microbial fermentation of feed or feed stuffs with a relatively high concentration of D-Lactate, such as silage. However, the endogenous production of D-lactate in human cells (and now ovine rumen epithelium) can result from the MGO pathway [36]. From *in vitro* studies it is clear bovine tissues possess D-lactate dehydrogenase (DLDH) [37] and we have evidence DLDH is increased in ovine epithelium tissue in the L CH_4_ sheep. Therefore D-lactate can be converted back to pyruvate by DLDH in the L CH_4_ epithelium and used in the TCA cycle to produce energy. In addition, the higher level of malic enzyme 1 in the L CH_4_ epithelium suggests these cells were also recycling malate from the TCA cycle to provide pyruvate for cellular energy transactions requiring NADH, FADH or ATP. Furthermore, resistin which is known to alter the sensitivity of cells to the hormonal control of blood glucose by insulin [38] had a higher abundance in the L CH_4_ sheep. Together the results provide evidence that the L CH_4_ sheep epithelium maybe more sensitive to glucose uptake from the rumen fluid and recycles intermediate metabolites during glycolysis to meet energy requirements of the cells unlike that found in the H CH_4_ epithelium.

Differences existed between the L and H CH_4_ epithelium in the abundance of proteins engaged in mechanisms to maintain immunity / cell defense against micro-organisms. In the H CH_4_ epithelium the abundance of nitric oxide synthase enzyme was higher than in the L CH_4_ epithelium. Cell wall components of microbia can lead to an immune response involving NOS [39]. Also, iron transporter SLC40A1 was higher abundance in the H than L CH_4_ emitting sheep which could be linked to a process called the Fenton reaction. Whereby iron (Fe^2+^) catalyses the conversion of hydrogen peroxide (H_2_O_2_), a product of mitochondrial oxidative respiration or processes in the peroxisome [20], into a highly toxic hydroxyl free radical [40]. Thereby facilitating the action of H_2_O_2_ to kill bacteria in the epithelium. In contrast, in the L CH_4_ epithelium myeloperoxidase was more abundant. Some phagocytes have the capacity to secrete enzymes called myeloperoxidases that can catalyse a reaction of H_2_O_2_ and halides such as chloride to produce hypochlorous acid (HOCl) [41, 42]. These hypohalous acids kill bacteria but can also damage normal tissue and thereby contribute to an inflammatory reaction. Also adaptor protein (AP) complex subunit beta had higher abundance in the L CH_4_ epithelium. Adaptor protein complexes function in a process by which cells absorb metabolites, hormones, proteins and in some cases viruses by the inward budding of the plasma membrane (invagination). Lastly the L CH_4_ epithelium had a greater abundance of an antimicrobial called Azurocidin 1 [43].

## Conclusion

Using our proteomics approach to quantify differences in protein abundance of rumen epithelium and related metabolites in the rumen fluid or blood in L or H CH_4_ emitting sheep has created new insights into the metabolic fate and metabolism of nutrients in the rumen epithelium. A protein transporter (SLCO2A1) previously not identified involved in SCFA and chloride transport was found. The main differences in protein abundance found between L or H CH_4_ emitting sheep were related to the metabolism of glucose. In addition, we found evidence the immune mechanism epithelium use in response to microbes was different in the L or H CH_4_ phenotype.

## Supporting information

S1 Table Proteomics data. The file summarizes the proteins identified and quantified in rumen epithelium of H and L CH_4_ emitting sheep. It includes the following data for protein identifications in separate worksheets 1) high pH fractionation by HPLC IDA-MS: protein and gene identifiers, name, ProtScore, confident peptides, % sequence coverage, Mass (Da), Gene ontology terms, subcellular location prediction 2) Transporter: protein and gene identifiers, gene name, solute carrier type, ProtScore, confident peptides, % sequence coverage 3) Key for SWATH-MS identification sheets 4) Cytosol proteins quantified by SWATH-MS 5) Membrane proteins quantified by SWATH-MS.
(XLS)

S2 Table Rumen fluid and blood metabolites data. (XLSX)

## Abbreviations

LC-MS/MS: Liquid chromatography-tandem mass spectrometry
HpH: high pH HPLC
IDA: independent data analysis
DIA: data-Independent Acquisition
SWATH-MS: sequential window acquisition of all theoretical mass spectra
FDR: false discovery rate
CT: computer tomography X–ray
RC: respiration chambers
GH: greenhouse gases
CH_4_: methane
CO_2_: carbon dioxide
DMI: dry matter intake
RFI: residual feed intake
LWT: liveweight
DMD: dry matter digestibility
MRT: mean retention time
SCFA: short chain fatty acids
AA: amino acids
BHB: beta−hydroxy butyrate
CP: crude protein
NDF: neutral detergent fibre
MJ ME /kg DM: megajoules of metabolisable energy per kg of dry matter
Tris: tris(hydroxymethyl)aminomethane
DTT: dithiothreitol
TEAB: triethylammonium bicarbonate
IAA: iodoacetamide
TMHMM: transmembrane helical Markov model
SignalP: signal peptide
ER: endoplasmic reticulum
SLC: solute carrier protein
ABC: ATP-binding cassette
NMR: nuclear magnetic resonance spectrometry
DSS: 4,4‑dimethyl‑4‑silapentane-1-sulfonic acid
DFTMP: difluorotrimethylsilylmethylphosphonic acid
ANOVA: analysis of variance
Na^+^: sodium
H^+^: hydrogen
K^+^: potassium
Cl^-^: chloride
Zn^2+^: zinc
Ca^2+^: calcium
NH_3_: ammonia
N: nitrogen
C: carbon
Fe^2+^: iron
NADH: nicotinamide adenine dinucleotide hydrogen
FADH: flavin adenine dinucleotide hydrogen
ATP: adenosine triphosphate
NOS: nitric oxide synthase
H_2_O_2_: hydrogen peroxide
HOCl: hypochlorous acid

## References

[1] Bond JJ, Cameron M, Donaldson AJ, Austin KL, Harden S, Robinson DL, et al. Aspects of digestive function in sheep related to phenotypic variation in methane emissions. Anim Prod Sci 2017; 59: 55–65.

[2] Pinares-Patiño CS, Hickey SM, Young EA, Dodds KG, MacLean S, Molano G, et al. Heritability estimates of methane emissions from sheep. Animal 2013; 7: 316–321. doi: 10.1017/S1751731113000864 23739473 PMC3691003

[3] Robinson DL, Goopy JP, Donaldson AJ, Woodgate RT, Oddy VH and Hegarty RS. Sire and liveweight affect feed intake and methane emissions of sheep confined in respiration chambers. Animal 2014; 8: 1935–1944. doi: 10.1017/S1751731114001773 25404195 PMC4255326

[4] Blaxter KL and Clapperton JL. Prediction of the amount of methane produced by ruminants. Brit J Nutr 1965; 19: 511–522. doi: 10.1079/bjn19650046 5852118

[5] Jonker A, Hickey S, Boma P, Woju CW, Sandoval E, Maclean S, et al. Individual-level correlations of rumen volatile fatty acids with enteric methane emissions for ranking methane yield in sheep fed fresh pasture. Anim Prod Sci, 2020; 61: 300–305.

[6] Graham C and Simmons NL. Functional organization of the bovine rumen epithelium. Am J Physiol Regul Integr Comp Physiol. 2005; 288: R173–R181. doi: 10.1152/ajpregu.00425.2004 15319221

[7] Jiang Y, Xie M, Chen W, Talbot R, Maddox JF, Faraut T, et al. The sheep genome illuminates biology of the rumen and lipid metabolism Science 2014; 344: 1173–1168. 24904168 10.1126/science.1252806 PMC4157056

[8] Bond JJ, Donaldson AJ, Coumans JVF, Austin K, Ebert D, Wheeler D, et al. Protein profiles of enzymatically isolated rumen epithelium in sheep fed a fibrous diet. J Anim Sci and Biotech. 2019; 10: 5. doi: 10.1186/s40104-019-0314-0 30697422 PMC6346531

[9] Kamath KS, Krisp C, Chick J, Pascovici D, Gygi SP and Molloy MP. Pseudomonas aeruginosa proteome under hypoxic stress conditions mimicking the cystic fibrosis lung. J Proteome Res. 2017; 16: 3917–3928. doi: 10.1021/acs.jproteome.7b00561 28832155

[10] Horton P, Park K-J, Obayashi T, Fujita N, Harada H, Adams-Collier CJ, et al. WoLF PSORT: protein localisation predictor. Nucleic acids res. 2007; 35: W585–W587. 17517783 10.1093/nar/gkm259 PMC1933216

[11] Krogh A, Larsson B, von Heijne G, Sonnhammer EL. Predicting transmembrane protein topology with a hidden Markov model: application to complete genomes. J Mol Biol. 2001; 305: 567–80. doi: 10.1006/jmbi.2000.4315 11152613

[12] Ross EM, Hayes BJ, Tucker D, Bond J, Denman SE, Oddy VH. Genomic predictions for enteric methane production are improved by metabolome and microbiome data in sheep (*Ovis aries*). J Anim Sci. 2020; 98: 262. 10.1093/jas/skaa262 PMC7751162 32815548

[13] McMurray C.H., Blanchflower W.J. and Rice D.A. Automated kinetic method for D-3-hydroxybutyrate in plasmas or serum. Clin Chem. 1984; 30: 421–425. 6697489

[14] Methods of Biochemical Analysis and Food Analysis, Boehringer Mannheim Publication,1989, pp.72–75.

[15] Wu JX, Song X, Pascovici D, Zaw T, Care N, Krisp C, et al. SWATH Mass Spectrometry Performance Using Extended Peptide MS/MS Assay Libraries. Mol Cell Proteom. 2016; 15: 2501–14. doi: 10.1074/mcp.M115.055558 27161445 PMC4937520

[16] Bjelosevic S, Pascovici D, Ping H, Karlaftis V, Zaw T, Song X, et al. Quantitative Age-specific Variability of Plasma Proteins in Healthy Neonates, Children and Adults. Mol Cell Proteom. 2017; 16: 924–935. doi: 10.1074/mcp.M116.066720 28336724 PMC5417830

[17] Pascovici D, Handler DC, Wu JX, Haynes PA. Multiple testing corrections in quantitative proteomics: A useful but blunt tool. Proteomics. 2016; 16: 2448–53. doi: 10.1002/pmic.201600044 27461997

[18] Minitab Statistical Software. [Computer software]. State College, PA: Minitab, Inc. (www.minitab.com). 2010.

[19] Sabirov RZ. Merzlyak PG, Okada T, Islam R, Uramoto H, Mori T, et al. The organic anion transporter SLCO2A1 constitutes the core component of the Maxi-Cl channel. EMBO J 2017; 36: 3309–3324. doi: 10.15252/embj.201796685 29046334 PMC5686547

[20] Wander RJA and Waterman HR. Biochemistry of mammalian peroxisomes revisited. Annu. Rev. Biochem. 2006; 75: 295–332. doi: 10.1146/annurev.biochem.74.082803.133329 16756494

[21] Georgi M-I, Rosendahl J, Ernst F, Günzel D, Aschenbach JR, Martens H, et al. Epithelia of the ovine and bovine forestomach express basolateral maxi-anion channels permeable to the anions of short chain fatty acids. Pflugers arch–Eur J Physiol. 2014; 466: 1689–1712. 24240698 10.1007/s00424-013-1386-x

[22] Stumpff F. A look at the smelly side of physiology: transport of short chain fatty acids. Pflugers arch–Eur J Physiol. 2018; 470: 571–598. 29305650 10.1007/s00424-017-2105-9

[23] Graham C, Gatherer I, Haslam I, Glanville M and Simmons NL. Expression and localisation of monocarboxylate transporters and sodium/proton exchangers in bovine rumen epithelium. Am. J Physiol Regul Integr Comp Physiol 2007; 292: R997–R1007. 17008462 10.1152/ajpregu.00343.2006

[24] Müller F, Huber K, Pfannkuche H, Aschenbach JS, Breves G, Gäbel G, Transport of ketone bodies and lactate in the sheep ruminal epithelium by monocarboxylate transporter 1. Am J Physiol-Gastr L. 2002; 283: G1139–1146. doi: 10.1152/ajpgi.00268.2001 12381528

[25] Okada Y, Okada T, Islam R and Sabirov RZ. Molecular identities and ATP release activities of two types of volume-regulatory anion channels, VSOR and Maxi-Cl. Curr Top Membr. 2018; 81: 125–176. doi: 10.1016/bs.ctm.2018.07.004 30243431

[26] Jentsch TJ, Lutter D, Planells-cases R, Ullrich F and Voss FK. VRAC: molecular identification as LRRC8 heteromers with differential functions. Pflugers arch–Eur J Physiol. 2016; 468: 385–393. doi: 10.1007/s00424-015-1766-5 26635246

[27] Schrapers KT, Sponder G, Leibe F, Liebe H and Stumpff F. The bovine TRPV3 as a pathway for the uptake of Na+, Ca2+ and NH4. PLos ONE 2018; 13: e0193519. doi: 10.1371/journal.pone.0193519 29494673 PMC5832270

[28] Launay P, Fleig A, Perraud AL, Scharenberg AM, Penner R, Kinet JP. TRPM4 is a Ca^2+^-activated nonselective cation channel mediating cell membrane depolarization. Cell. 2002; 109: 397–407. 12015988 10.1016/s0092-8674(02)00719-5

[29] Bergman EN. Energy contributions of volatile fatty acids from the gastrointestinal tract in various species. Physiol Rev. 1990; 70: 567–590. doi: 10.1152/physrev.1990.70.2.567 2181501

[30] Saleem F, Bouatra S, Guo AC, Psychogios N, mandal R, Dunn SM, et al. The bovine ruminal fluid metabolome. Metabolomics 2013; 9: 360–378.

[31] Ostrowska M, Jarczak J and Zwierzchowski L. Glucose transporters in cattle—A review. Anim sci pap. 2015; 33: 191–212.

[32] Aschenbach JR, Bhatia SK, Pfannkuche H, Gäbel G. Glucose is absorbed in a sodium-dependent manner from forestomach contents of sheep. J Nutr. 2000; 130: 2797–2801 doi: 10.1093/jn/130.11.2797 11053523

[33] Goopy JP, Donaldson A, Hegarty R, Vercoe PE, Haynes F, Barnett M, et al. Low-methane sheep have smaller rumens and shorter rumen retention time. Brit J Nutrition 2014; 111: 578–85. 24103253 10.1017/S0007114513002936

[34] Pinares-Patiño CS, Ulyatt MJ, Lassey KR, Barry TN and Holmes CW. Rumen function and digestion parameters associated with differences between sheep in methane emissions when fed chaffed Lucerne hay. J. Agric. Sci. 2003; 140: 205–214.

[35] Kamke J, Kittlemann S, Soni P, Li Y, Tavendale M, Ganesh S, et al. Rumen metagenome and metatranscriptome analyses of low methane yield sheep reveals a *Sharpea*-enriched microbiome characterised by lactic acid formation and utilisation. Microbiome. 2016; 4: 56. 27760570 10.1186/s40168-016-0201-2 PMC5069950

[36] Ewaschuk JB, Naylor JM and Zello GA. D-Lactate in Human and Ruminant Metabolism J. Nutr. 2005; 135: 1619–1625. doi: 10.1093/jn/135.7.1619 15987839

[37] Harmon D. L., Britton R. A. & Prior R. L. *In vitro* rates of oxidation and gluconeogenesis from L(+)- and D(-)lactate in bovine tissues. Comp. Biochem. Physiol. B. 1984; 77: 365–368. 6697693 10.1016/0305-0491(84)90344-4

[38] Pang S and Le Y. Role of resistin in inflammation and inflammation related diseases. Cell Mol Immunol. 2006; 3: 29–34. 16549046

[39] Dawson TM and Snyder SH. Gases as biological messengers: nitric oxide and carbon monoxide in the brain. J Neurosci. 1994; 1: 5147–59. 10.1523/JNEUROSCI.14-09-05147.1994 PMC6577075 8083727

[40] Galaris D, Barbouti A and Pantopoulos K. Iron homeostasis and oxidative stress: An intimate relationship. Biochim Biophys Acta Mol Cell Res. 2019; 1866: 118535. doi: 10.1016/j.bbamcr.2019.118535 31446062

[41] Winterbourn CC, Hampton MB, Livesey JH and Kettle AJ. Modelling the Reactions of Superoxide and Myeloperoxidase in the Neutrophil Phagosome. Implications for microbial killing. J Biol Chem. 2006; 281: 39860–39869. 17074761 10.1074/jbc.M605898200

[42] Bakkenist ARJ, De Boer JEG, Plat H and Wever R. The halide complexes of myeloperoxidase and the mechanism of the halogenation reactions. Biochim Biophys Acta. 1980; 613: 337–348. doi: 10.1016/0005-2744(80)90088-1 6255998

[43] Kościuczuk E.M, Lisowski P, Jarczak J, Strzałkowska N, Jóźwik A, Horbańczuk J, et al. Cathelicidins: family of antimicrobial peptides. A review. Mol. Biol. Rep. 2012; 39: 10957–10970. doi: 10.1007/s11033-012-1997-x 23065264 PMC3487008

